# Determination of Key Environmental Factors Responsible for Distribution Patterns of Fiddler Crabs in a Tropical Mangrove Ecosystem

**DOI:** 10.1371/journal.pone.0117467

**Published:** 2015-01-28

**Authors:** Mohammad Mokhtari, Mazlan Abd Ghaffar, Gires Usup, Zaidi Che Cob

**Affiliations:** 1 School of Environmental and Natural Resource Sciences, Faculty of Science and Technology, National University of Malaysia, 43600 UKM Bangi, Selangor D.E., Malaysia; 2 Marine Ecosystem Research Centre, Faculty of Science and Technology, National University of Malaysia,43600 UKM Bangi, Selangor D.E., Malaysia; University of Western Australia, AUSTRALIA

## Abstract

In tropical regions, different species of fiddler crabs coexist on the mangrove floor, which sometimes makes it difficult to define species-specific habitat by visual inspection. The aim of this study is to find key environmental parameters which affect the distribution of fiddler crabs and to determine the habitats in which each species was most abundant. Crabs were collected from 19 sites within the mudflats of Sepang-Lukut mangrove forest. Temperature, porewater salinity, organic matter, water content, carbon and nitrogen content, porosity, chlorophyll content, pH, redox potential, sediment texture and heavy metals were determined in each 1 m^2^ quadrate. Pearson correlation indicated that all sediment properties except pH and redox potential were correlated with sediment grain size. Canonical correspondence analysis (CCA) indicated that *Uca paradussumieri* was negatively correlated with salinity and redox potential. Sand dwelling species, *Uca perplexa* and *Uca annulipes*, were highly dependent on the abundance of 250 μm and 150 μm grain size particles in the sediment. Canonical Discriminative Analysis (CDA) indicated that variation in sediment grain size best explained where each crab species was most abundant. Moreover, *U. paradussumieri* commonly occupies muddy substrates of low shore, while *U. forcipata* lives under the shade of mangrove trees. *U. annulipes* and *U. perplexa* with the high number of spoon tipped setae on their second maxiliped are specialized to feed on the sandy sediments. *U. rosea* and *U. triangularis* are more common on muddy sediment with high sediment density. In conclusion, sediment grain size that influences most sediment properties acts as a main factor responsible for sediment heterogeneity. In this paper, the correlation between fiddler crab species and environmental parameters, as well as the interaction between sediment characteristics, was explained in order to define the important environmental factors in fiddler crab distributions.

## Introduction

Fiddler crabs are common benthic macrofauna in intertidal mudflats of mangrove forest of south-east Asia [1]. These crabs stay near their burrows into which they can easily escape from predators, as well as refuge from high temperatures and desiccation [2]. Fiddler crabs have high tolerance to low oxygen level, high temperatures and varying salinities [3]. From 97 identified species of fiddler crabs, eight species have been recorded from west Peninsular Malaysia [4,5]. In the study area, seven species of fiddler crabs including *Uca Paradussumieri*, *Uca rosea*, *Uca trinagularis*, *Uca forcipata*, *Uca perplexa*, *Uca annulipes* and *Uca vocans were* distributed unevenly among the intertidal mudflats. There were some degrees of overlap between species territories as their habitats were not visually discernible.

Predation, recruitment and sediment heterogeneity are the main factors determining the distribution pattern of benthic communities. Since the factors that determine the distribution pattern of fiddler crabs can differ between adults and juveniles [6], the recruitment pattern may not determine the spatial distribution of adults. Following settlement, juvenile crabs may change their habitat according to their changing habitat requirements as they grow. For instance, juvenile *U*. *pugilator* feed on the muddy substrates and lack the specialized mouthpart structures needed to feed efficiently on sandy sediments like adults [7,8]. Hogarth [3] claimed that distribution pattern of fiddler crabs is the result of interaction between physiological stress, predation risk, feeding preference and social behavior. A variety of predators including birds and fishes feed on fiddler crabs in mangrove forests. By hiding in their burrows, fiddler crabs escape from the predation and therefore will survive in areas where they are able to excavate burrows. Moreover, Bertrness and Miller [9] stated that factors that affect the ability of fiddler crabs to dig burrows are important in the determination of crab distributions. The size of the crabs is important in determining their predation risk as it was documented that some small avian predators select small crabs and avoid large individuals [10]. In this study, except for large male of *U*. *paradussumieri*, fiddler crab species possess a similar range of body sizes (*U*. *rosea*: 5.5–22.5mm, *U*. *forcipata*: 6–23mm, *U*. *paradussumieri*: 9–30mm, *U*. *triangularis*: 5–15.5mm, *U*. *perplexa*: 6–16, *U*. *annulipes*: 7.5–18.2, *U*. *vocans*: 10.5–23.5). Hence, because these crabs are so similar in size, significant differences in predation rate between these species are unlikely. Even though different predation rate exists, it is unlikely to expect that predators differentially remove one species from a given habitat.

Spatial variation in sediment properties and heterogeneous characteristics of mangrove sediment create different niches for different species to occupy. Some species prefer fine grained sediments, while others tend to occupy coarse sandy substrates. Fiddler crabs that occupy sandy sediments possess higher numbers of spoon tipped setae on their second maxiliped and these setae are believed to hold sand grains during feeding activity and aid in removal and sorting organic particles (e.g., bacteria) from the grains of sand [7]. Fiddler crabs consume bacteria, diatoms, ciliated protozoa, and nematodes and they tend to reject large algal cells and mangrove detritus [3,11]. Another explanation of spatial distribution of fiddler crabs is the fact that crabs tend to occupy and feed on the rich mudflats to increase their intake efficiency [12].

There have been many studies of the factors that affect the spatial distributions of fiddler crabs. Some researchers measured fiddler crab tolerances to different salinities and temperatures in the laboratory [1,13]. Bezerra et al., [14] measured the relation between the abundance of each species and amount of organic matter, water content, sediment texture and the presence of other species. The previous studies [1,7,13,14] have shown that sediment texture is important in distribution pattern of fiddler crabs so that some species live on sandy sediments, while others tend to occupy muddy substrates. However, the correlation between environmental parameters especially the sediment grain size and fiddler crab community has not been quantified yet. The main purpose of the current study is to correlate the crab distribution pattern with sediment properties. To the best of our knowledge, no attempt has been made to correlate the environmental factors with fiddler crab community using multivariate techniques. Accordingly, this research by the use of multivariate analysis such as canonical corresponding analysis (CCA) and Canonical discriminant analysis (CDA) aims to find key environmental parameters responsible for spatial distribution of fiddler crabs and define the favorable range of these parameters for different species of fiddler crabs.

## Materials and Methods

### Ethics Statement:

Field sampling near the power plant was conducted with the permission #002020 obtained from Jimah Energy Ventures Sdn Bhd. This study did not involve protected or endangered species.

### Study area:

Field sampling was conducted in Sepang-Lukut fringing mangrove forests around the river mouth of Sungai Sepang Besar, west coast of Peninsular Malaysia. Tides are semidiurnal with high amplitude (>2.5 m). Fiddler crabs were distributed from low tide to the high tide levels on the creek’s mud banks.

### Sampling procedure:

**Fig 1:**
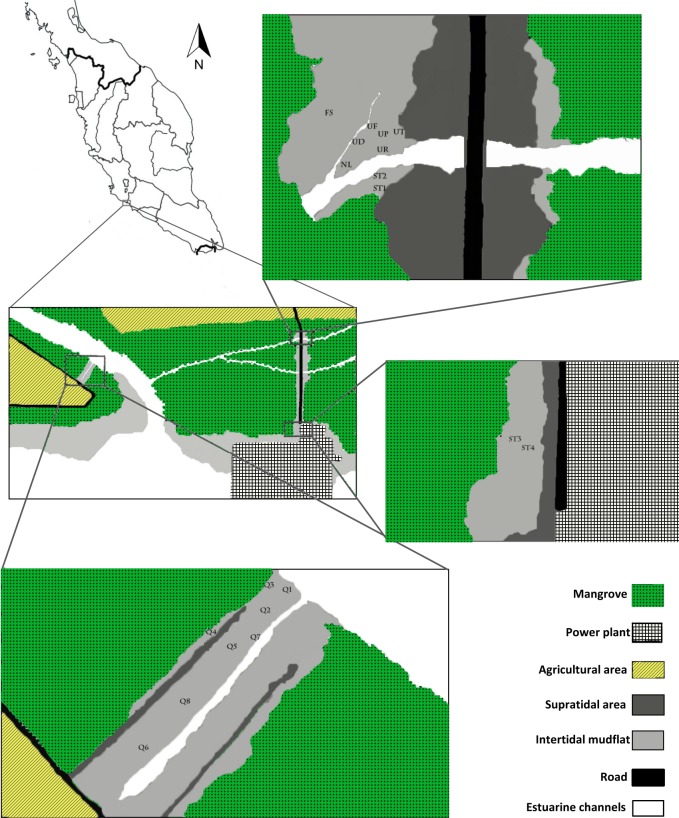
The position of sampling stations among three study areas. UP- *Uca perplexa* site (02° 36’ 07.9” N/ 101° 43’ 23.2” E); UD- *Uca paradussumieri* site (02° 36’ 07.7” N/ 101°43’ 22.9” E); UT- *Uca triangularis* site (02° 36’07.8” N/ 101° 43’ 23.3” E); UR- *Uca rosea* site (02° 36’ 07.6” N/ 101° 43’ 23.3” E); UF- *Uca forcipata* site (02° 36’ 07.9” N/ 101° 43’ 23.1” E); FS- forest site (02° 36’ 08.1” N/ 101° 43’ 22.9” E); NL- low shore site (02° 36’ 07.4”/ 101° 43’ 22.9” E); ST1- station 1 (02° 36’ 07.1” N/ 101° 43’ 23.2” E); ST2- station 2 (02° 36’ 07.3” N/ 101° 43’ 23.2” E); ST3- station 3 (02° 35’ 44.7” N/ 101° 43’ 23.1” E); ST4- station 4 (02° 35’ 44.5” N/ 101° 43’ 23.3” E); Q1 (02° 35’ 59.9” N/ 101° 42’ 31.6” E); Q2 (02° 35’ 59.2” N/ 101° 42’ 31.2” E); Q3 (02° 35’ 59.8” N/ 101° 42’ 31.1” E); Q4 (02° 35’ 58.5” N/ 101° 42’ 30.2” E); Q5 (02° 35’ 58.3” N/ 101° 42’ 30.7” E); Q6 (02° 35’ 56.3” N/ 101° 42’ 29.6” E); Q7 (02° 35’ 58.5” N/ 101° 42’ 31.1” E); Q8 (02° 35’ 57.3” N/ 101° 42’ 30.1” E).

Sampling stations were chosen according to the fiddler crab occurrence, species composition, mangrove trees, shore level and sediment texture. Samples were collected from 19 stations, with three replications of 1 m^2^ quadrate each (Fig. 1). Burrow density within the quadrate was counted and subsequently redox potential, pH and temperature of the surface sediment were measured in situ by ORP meter (Exstick Model RE 300) and pH meter (Exstick Model pH 100), respectively. Porewater salinity was measured by refractometer after collecting interstitial water by digging holes into the sediment. For determination of chlorophyll content, a thin layer (3mm) of surface sediment was scraped by steel spatula, packed in zipped plastic bags, labeled and stored in ice box until laboratory measurement within 24 hours. Surface sediment was collected (2cm thickness) and kept in plastic bags for determination of sediment properties. The remainders of sediments in the quadrates were dug with a shovel and the crabs were collected by hand from the excavated sediments. Fiddler crabs were preserved in 70% ethanol immediately after collection and were identified later in laboratory.

### Laboratory analysis:

To estimate the number of spoon tipped setae in second maxillipeds, a group of individuals from each species was selected. The carapace width of selected crabs was measured with Vernier caliper. The left second maxillipeds were removed with fine forceps and spoon tipped setae’s were enumerated under stereo microscope. Since the number of spoon tipped setae is a function of body size, it was regressed against carapace width and crabs with 15 mm carapace width were used as an index to compare the number of spoon tipped setae between species.

In laboratory parameters attributed with the sediment such as grain size, density, water content, porosity, organic content, and total carbon and nitrogen content were determined. The sediment grain size was defined by dry sieving method [16]. Sediment density was determined by weighing a known volume of sediment whilst water content was measured by weight loss after drying at 70°C for 12 hours. Using the density and water content values the sediment porosity was then calculated using the following formula: (density × water content)/100. The sediment organic content was estimated by loss on ignition method at 520 ° C for 12 hours, and finally the sediment total carbon and nitrogen were measured by PerkinElmer CHNS elemental analyzer.

Apart from that other parameters i.e. the chlorophyll content and heavy metals were also analyzed. The chlorophyll contents were measured by the Lorenzen method [15]. About 0.5 g of sediment were incubated overnight in 90% acetone at 4̊C followed by centrifugation at 3000 rpm for 5 min. The chlorophyll content was subsequently determined by spectrophotometer at 665 and 750 nm before and after acidification with 1 M HCL. The heavy metal such as Copper (Cu), Zinc (Zn), Lead (Pb), Nickel (Ni), Cadmium (Cd) and Chromium (Cr) concentrations were quantified by ICP-mass spectrometer (Perkin Elmer Sciex). Prior to analysis, dry sediment samples were digested by nitric acid-perchloric acid method following the procedures explained in APHA [17].

### Data analysis:

Pearson correlation was performed to determine the possible correlation between environmental parameters and burrow density. Non-metric multi-dimensional scaling (nMDS) [18] and cluster analysis were applied to describe the similarity between the sampling stations using PRIMER—E statistical software version 5 [19]. To correlate the environmental parameters with crab community structure, Canonical Corresponding Analysis (CCA) [20] through CANOCO package version 4.5 [21] were performed. Accordingly, key environmental parameters were determined by Canonical discriminant analysis (CDA) by means of STATISTICA package version 11 [22].

## Results

**Fig 2:**
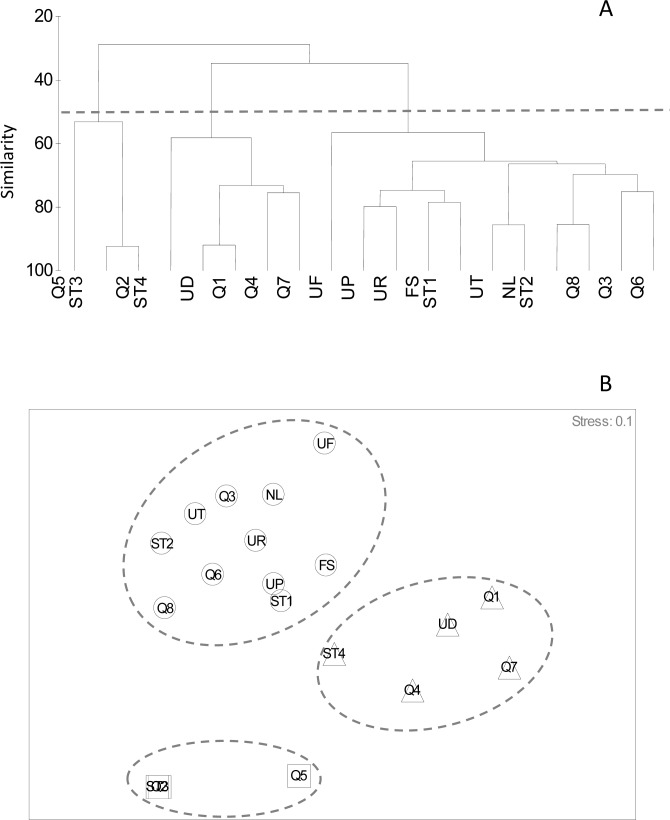
Cluster dendogram (a) and nMDS graph (b) of sampling stations based on crab species composition. Sampling sites were divided into the three groups at level of 50% bray-Curtis similarity. Triangle (Δ) = *U*. *paradussumieri* dominant sites, Square (□) = sandy sites, Circles (○) = station where *U*. *rosea* and *U*. *triangularis* are common.

**Fig 3:**
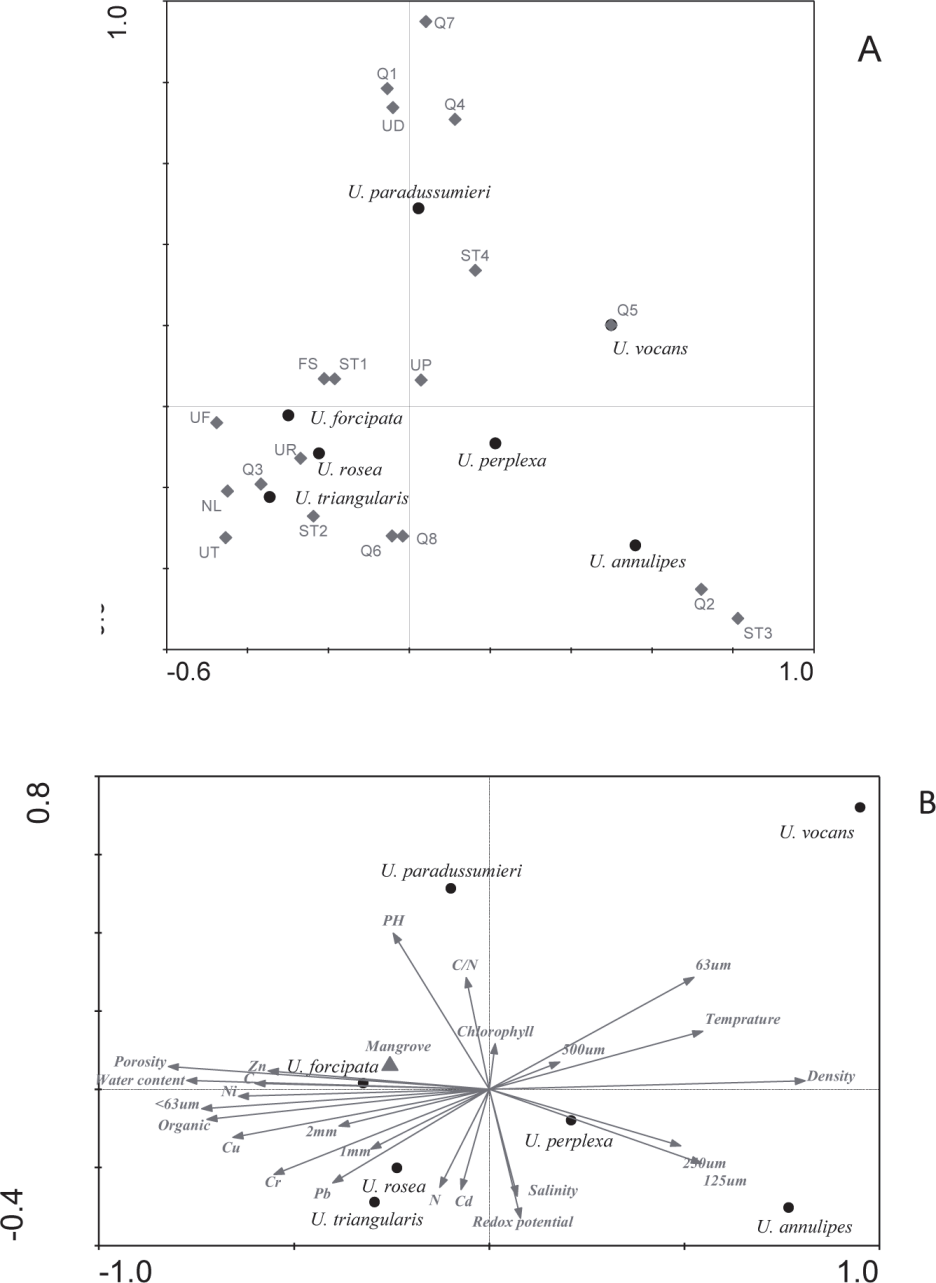
CCA results including biplot of species versus station (a) and species versus environmental parameters (b). Circle (●) and diamond (◊) symbols indicate the position of sampling stations and species respectively, while environmental factors are represented by triangle (Δ) and arrows.

Different species of fiddler crabs were distributed unevenly among stations (Table 1). *U*. *perplexa* was the most common species, which was observed in 16 stations, while *U*. *vocans* was found only in one location. Crabs territory was highly overlapped as showed by the Pianka’s overlap index (Table 2). Almost all species coexist with each other except for *U*. *vocans*, which did not share its territory with *U*. *rosea*, *U*. *forcipata*, and *U*. *triangularis* (Table 2). Cluster analysis indicated that crab community was not significantly different between the three study areas (Fig. 2A), suggesting that fine scale variability like sediment heterogeneity is responsible for changes in crab community composition. At 50 percent similarity levels, three groups of stations were observed. The first group includes Q2, ST3 and Q5 sampling stations. In these stations, the amount of sand particles was higher than those in other groups; therefore were labeled as sandy group in the nMDS graph (Fig. 2B). Stations Q2 and ST3 which were located on the opposite sides of the river mouth showed high similarity due to the presence of *U*. *annulipes* and *U*. *perplexa* as dominant species. The second group (Q1, St4, Q4, UDH and Q7) comprised of stations that where *U*. *paradussumieri* was dominant. The remaining sampling stations (ST2, Q3, UT, NL, UF, FS, UR, UP, ST1, Q6 and Q8) formed the third group where *U*. *rosea* and *U*. *triangularis* were the two most frequent species. The results of one-way ANOVA suggest that all environmental parameters and crab densities were significantly different among the stations (p < 0.05). Further analysis using CCA (Fig. 3A) displayed a close relationship between species and their actual habitats as showed by Fig. 2. *U*. *paradussumieri* and *U*. *rosea* were located very close to UDH and URH, respectively. In the biplot of species and environmental variables, *U*. *perplexa* and *U*. *annulipes* were more dependent on sand particles (250 and 125 μm) than other environmental factors (Fig 3B). *U*. *rosea* and *U*. *triangularis* were positively correlated with high concentrations of nitrogen content and negatively correlated with very fine sand particles (63 μm). *U*. *forcipata* which was observed near mangrove trees during the field sampling shows a high dependency on fine grained sediments as well as a positive correlation with porosity and organic, carbon and water content. Fig. 3B also showed that *U*. *paradussumieri* is positively correlated with pH and C:N ratio and negatively correlated with pore water salinity and redox potential.

**Table 1:** The percentage of seven species of fiddler crabs among sampling stations.

	U. paradussumieri	U. rosea	U. triangularis	U. forcipata	U. perplexa	U. annulipes	U. vocans
UT	3	19	68	0	10	0	0
UP	24	17	10	4	45	0	0
UF	10	10	20	60	0	0	0
UR	11	33	28	8	20	0	0
UD	79	0	0	17	4	0	0
NL	12	16	62	5	5	0	0
FS	21	42	0	21	16	0	0
ST1	25	57	4	0	14	0	0
ST2	0	42	29	0	29	0	0
ST3	0	0	0	0	29	71	0
ST4	53	12	0	6	12	17	0
Q1	83	0	0	17	0	0	0
Q2	0	0	0	0	44	56	0
Q3	0	20	23	40	17	0	0
Q4	79	0	0	0	21	0	0
Q5	24	0	0	0	39	9	28
Q6	3	15	31	13	16	22	0
Q8	0	39	19	0	27	15	0
Q7	100	0	0	0	0	0	0

**Table 2:** Pianka's index of habitat overlap.

	UD	UR	UT	UF	UP	UA	UV
UD	1	0.257	0.177	0.191	0.378	0.091	0.334
UR	0.257	1	0.617	0.428	0.406	0.093	0
UT	0.177	0.617	1	0.365	0.244	0.063	0
UF	0.191	0.428	0.365	1	0.219	0.05	0
UP	0.378	0.406	0.244	0.219	1	0.663	0.671
UA	0.091	0.093	0.063	0.05	0.663	1	0.197
UV	0.334	0	0	0	0.671	0.197	1

UD = *U*. *paradussumieri* habitat, UR = *U*. *rosea* habitat, UT = *U*. *traingularis* Habitat, UF = *U*. *forcipata* habitat, UP = *U*. *perplexa* habitat, UA = *U*. *annulipes* habitat, UV = *U*. *vocans* habitat.

**Fig 4:**
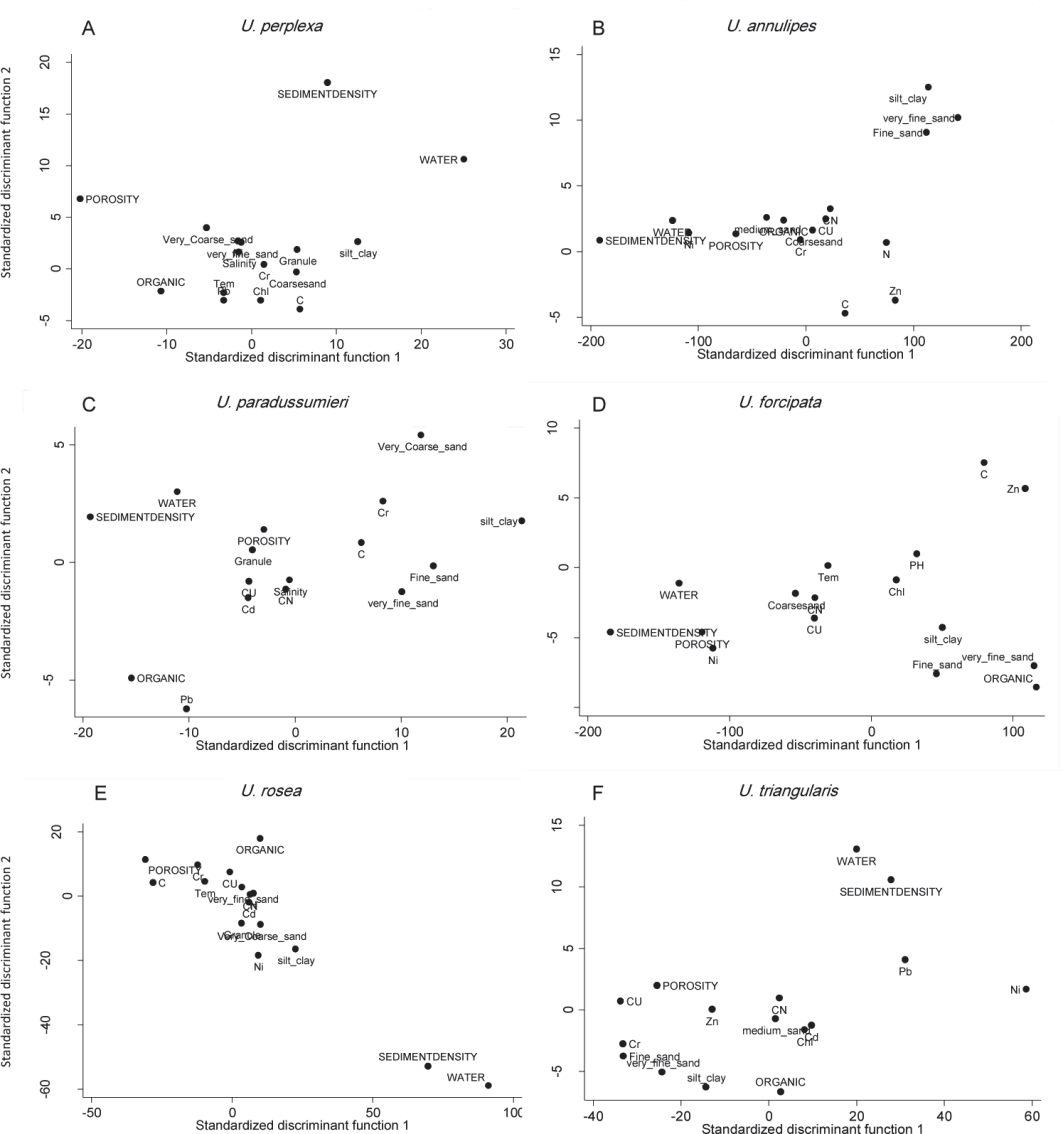
Standardized discriminant function plot for different species of fiddler crabs. Environmental variable located on the edge of plot are significantly different between abundant, rare and absence sites and therefore plays important role in determination of crab habitat.

To determine fiddler crabs habitat, CDA analyses were performed for each species separately. Prior to analysis, the sampling stations were classified into three groups based on crab abundance. Stations that host more than four individuals from the respective species were categorized as abundant group. Accordingly, stations with less than four crabs were classified as rare, and stations where the relevant species were not found were labeled as absent group. By performing CDA, a linear function was calculated from a combination of environmental variables that can best separate these three groups. A total of 19 stations and 25 environmental variables were used to create the discriminant functions. For *U*. *paradussumieri*, the most discriminant variables by the order of importance were water content, sediment density, porosity, organic content, Cd, carbon content, salinity, fine sand and silt-clay content (Fig. 4C). Canonical correlation coefficients and eigenvalue were significantly high for all the discriminant functions. Water content, sediment density, porosity, silt-clay, Ni, Pb, Cr and Cu, fine and very fine sand contents are recognized as the most discriminant factors and have important role in determining *U*. *triangularis* habitat (Fig. 4F). Water, organic and carbon content, sediment density, Cu and porosity are the most discriminant factors for determining *U*. *rosea* habitat (Fig. 4E). Sediment density, water content, porosity, organic and carbon content, very fine sand and the amount of Zn and Ni play an important role in determining *U*. *forcipata* habitat (Fig. 4D). Loading plots reveal that silt-clay, water content, porosity, fine sand, organic content and sediment density are more important in determining the *U*. *perplexa* habitat (Fig. 4A). However, silt-clay, fine sand and very find sand appeared to play much important role in determining *U*. *annulipes* habitat (Fig. 4B).

Almost all heavy metal elements were negatively correlated with fine and very fine sand and were positively correlated with silt-clay contents (Table 3). The significant correlation of heavy metals with sediment parameters including porosity, water and organic content, chlorophyll, temperature, and carbon content might be due to the relationship between silt-clay content and these parameters. Crab density was negatively correlated with chlorophyll content and sediment porosity, and positively correlated with medium sand (250 μm).

**Table 3:** Pearson correlation between burrow density and sediment properties.

	Dens	pH	Tem	RP	Sal	Chl	Poro	Wat	OM	N	C	C/N	2mm	1mm	500	250	125	63	<63	CU	Zn	Ni	Cd	Cr	Pb
Dens	1.00																								
pH	-0.229																								
Tem	0.165	0.152																							
RP	0.122	-0.23	0.029																						
Sal	0.015	0.155	0.032	-0.4																					
Chl	-0.26	0.412	0.172	-0.04	-0.03																				
poro	-0.289	0.065	-0.40	-0.09	-0.005	-0.07																			
Wat	-0.245	0.008	-0.41	-0.17	0.094	-0.143	0.979																		
OM	-0.129	-0.14	-0.39	-0.11	0.137	-0.41	0.857	0.898																	
N	-0.018	-0.17	-0.25	0.15	0.237	-0.42	-0.01	0.038	0.134																
C	-0.208	-0.14	-0.36	-0.17	0.155	-0.36	0.818	0.857	0.900	0.065															
C/N	-0.139	0.15	0.065	-0.10	-0.186	0.191	0.19	0.145	0.05	-0.65	0.30														
2mm	0.054	0.021	-0.28	-0.08	0.185	-0.24	0.279	0.353	0.44	0.079	0.32	-0.05													
1mm	0.147	0.043	-0.28	-0.10	0.302	-0.27	0.14	0.215	0.384	0.057	0.26	-0.08	0.91												
500μm	0.027	0.048	-0.29	-0.06	0.135	-0.31	-0.13	-0.04	0.12	0.21	0.06	-0.13	0.73	0.78											
250μm	0.370	-0.15	-0.23	-0.07	0.370	-0.15	-0.53	-0.44	-0.37	0.366	-0.30	-0.31	-0.096	0.05	0.28										
125μm	0.279	-0.18	0.031	0.06	0.279	-0.13	-0.65	-0.59	-0.54	0.430	-0.45	-0.31	-0.25	-0.13	0.14	0.86									
63μm	-0.114	0.24	0.435	-0.02	-0.253	0.581	-0.52	-0.57	-0.77	-0.41	-0.68	0.20	-0.42	-0.49	-0.30	-0.09	0.052								
<63μm	-0.139	-0.03	-0.22	0.01	-0.104	-0.25	0.827	0.800	0.868	-0.07	0.75	0.12	0.31	0.25	-0.06	-0.66	-0.78	-0.63							
CU	0.062	-0.10	-0.44	0.05	0.095	-0.34	0.649	0.669	0.808	0.12	0.70	0.012	0.48	0.52	0.27	-0.19	-0.42	-0.82	0.78						
Zn	0.018	0.024	-0.40	0.006	0.130	-0.28	0.592	0.604	0.659	0.18	0.64	0.07	0.17	0.22	0.12	-0.11	-0.28	-0.68	0.63	0.83					
Ni	-0.025	-0.07	-0.42	-0.04	0.113	-0.35	0.701	0.720	0.793	0.23	0.75	0.004	0.18	0.20	0.05	-0.17	-0.35	-0.76	0.74	0.87	0.94				
Cd	0.077	0.077	-0.32	0.08	0.209	-0.23	0.05	0.126	0.170	0.25	0.215	-0.05	0.45	0.47	0.49	0.23	0.018	-0.29	0.064	0.37	0.37	0.28			
Cr	-0.021	-0.12	-0.40	0.016	0.136	-0.34	0.485	0.513	0.700	0.16	0.54	-0.15	0.59	0.67	0.45	0.13	-0.34	-0.78	0.67	0.86	0.62	0.69	0.33		
Pb	0.005	-0.03	-0.24	0.05	0.108	-0.25	0.24	0.247	0.397	0.064	0.251	-0.14	0.63	0.69	0.52	-0.10	-0.24	-0.47	0.38	0.50	0.17	0.21	0.17	0.77	

Dens = burrow density, Tem = temperature, RP = Redox Potential, Sal = salinity, Chl = Chlorophyll content, poro = porosity, water = water content, OM = organic content, N = nitrogen content, C = carbon content, C/ N = C: N ratio, 2mm, 1mm, 500 μm, 250 μm, 125 μm, 63μm and <63 μm = sediment grain size particles

Significant correlations (p < 0.05) were underlined.

Silt-clay, fine sand and very fine sand are the most important factors in determining *U*. *annulipes* habitat. This species was most abundant on substrates with sediment texture of 32.45 ± 7.24% of very fine sand, 40.96 ± 9.24% of silt-clay, 18.45 ± 10.74% of fine sand (Table 4). *U*. *perplexa* chooses the substrates with 53.53 ± 8.08% silt-clay, 14.97 ± 6.23% of fine sand and 24.96 ± 6.03% of very fine sand and 0.56 ± 0.07% coarse sand. *U*. *forcipata* lives on sediments that contain 26.15 ± 17.68% of very fine sand, 0.43 ±0.01% of coarse sand, 65.63 ± 17.42% silt-clay and 4.87 ±0.87% of fine sand. Sediment texture was less important than other parameters in defining *U*. *rosea* habitat. *U*. *rosea* was commonly found on sediments with 72.07 ± 5.39% of silt-clay and 14.39 ± 4.75% of very fine sand. *U*. *triangularis* was more abundant on the substrates with 66.85 ± 7.26% of silt-clay, 17.82 ± 6.86% very fine sand, 9.00 ± 5.04% of fine sand and 1.81 ± 0.38% medium sand. *U*. *paradussumieri* commonly lives in fine grained sediments with 63.1 ± 7% silt-clay, 10 ± 2.7% fine sand and 21.4 ± 4.80% very fine sand. The CCA results showed that there was a positive relationship between pH and *U*. *paradussumieri* and a negative relationship between pH and *U*. *perplexa* (Fig 3B). However, the pH was an important factor in determining the *U*. *forcipata* habitat with optimum range of 7.22± 0.11.

**Table 4:** Optimum range of environmental variables for six species of fiddler crabs.

Discriminate variables	*U.paradussumieri*	*U.triangularis*	*U.rosea*	*U.forcipata*	*U.perplexa*	*U.annulipes*	*U.vocans*
pH	6.92 ± 0.2	6.86 ± 0.11	6.89 ± 0.11	7.22 ±0.11	6.88 ± 0.13	6.71± 0.13	6.86
Temperature (C)	31.88 ± 0.4	31.37 ± 0.4	31.47 ± 0.3	31.18±0.88	32.13 ± 0.39	32.4 ± 0.6	33.23
ORP (mV)	84.26 ±17.56	112.6 ± 8.9	88.51±15.86	95.33 ± 1	104.83±12.35	101.6±23.7	75.66
Salinity (PPT)	21.79 ± 1.5	24.33 ± 2.5	25.15 ± 1.9	26.1± 2.41	26.31 ± 2.04	24.75±3.76	22.66
Chl (μmol/g)	2.49 ± 0.56	2.72 ± 0.67	2.07 ± 0.54	4.42 ± 1.25	2.63 ± 0.55	2.74 ± 0.34	2.89
Sediment density (g/ml)	1.69 ± 0.1	8.07 ± 0.32	8.74 ± 0.27	7.78 ± 0.85	8.91 ± 0.32	9.47 ± 0.35	9.71
Porosity	2.8 ± 0.16	3.01 ± 0.15	3.10 ± 0.13	3.26 ± 0.33	2.60 ± 0.18	2.31 ± 0.25	2.30
Water content (%)	33.9 ± 3.1	37.96 ± 3.19	40.47 ± 3.13	42.89±8.99	30.18 ± 3.32	24.85±3.80	23.75
Organic content (%)	8.2 ± 1.6	9.91 ± 1.97	11.63 ± 1.63	11.37±6.13	6.68 ± 1.83	3.39 ± 0.97	3.46
N (%)	2 ± 1.15	3 ± 1.34	2.76 ± 0.93	2.18 ± 2.1	2.15 ± 1.13	0.91 ± 0.83	0.08
C (%)	2.78 ± 0.8	2.95 ± 0.82	4.04 ± 0.99	3.69 ± 2.69	1.94 ± 0.74	0.64 ± 0.23	0.61
C/N	9.32 ± 2.25	4.93 ± 1.99	7.49 ± 2.49	7.10 ± 5.58	7.2 ± 2.3	6.22 ± 2.05	8.17
2 mm (granule) (%)	1.2 ± 0.26	2.44 ± 1.16	2.47 ± 0.76	0.93 ± 0.48	1.11 ± 0.33	0.7 ± 0.26	0.53
1mm(very coarse sand) (%)	0.78 ± 0.2	1.46 ± 0.75	1.46 ± 0.5	0.65 ± 0.31	0.82 ± 0.23	0.53 ± 0.18	0.43
500 μm (coarse sand) (%)	0.61 ± 0.08	0.60 ± 0.12	0.58 ± 0.08	0.43 ± 0.01	0.56 ± 0.07	0.59 ± 0.13	0.74
250μm(medium sand) (%)	2.9 ±1	1.81 ± 0.38	1.72 ± 0.26	1.37± 0.29	4.05 ± 2.35	6.29 ± 4.70	3.04
125μm (fine sand) (%)	10 ± 2.7	9.00 ± 5.04	7.28 ± 3.38	4.87 ± 0.87	14.97 ± 6.23	18.45±10.74	14.13
63μm (very fine sand) (%)	21.4 ± 4.8	17.82 ± 6.86	14.39 ± 4.75	26.15±17.68	24.96 ± 6.03	32.45 ± 7.24	40.16
<63μm (silt & clay) (%)	63. 1 ± 7	66.85 ± 7.26	72.07 ± 5.39	65.63±17.42	53.53 ± 8.08	40.96 ± 9.24	40.96
Cu (PPM)	0.18 ± 0.03	0.20 ± 0.04	0.22 ± 0.03	0.21 ± 0.11	0.17± 0.03	0.11 ± 0.03	0.08
Zn (PPM)	1.31 ± 0.13	1.22 ± 0.12	1.32 ± 0.09	1.42 ± 0.40	1.18 ± 0.11	0.97 ± 0.07	1
Ni (PPM)	0.15 ± 0.02	0.13 ± 0.02	0.15 ± 0.02	0.18 ± 0.09	0.12 ± 0.02	0.083±0.015	0.08
Cd (PPM)	0.001 ± 0.0	0.002 ± 0.0	0.002 ± 0	0.001 ± 0.0	0.002 ± 0.0	0.002 ± 0.0	0.001
Cr (PPM)	0.36 ± 0.05	0.42 ± 0.12	0.044 ± 0.08	0.45 ± 0.23	0.32 ± 0.05	0.25 ± 0.05	0.22
Pb (PPM)	0.30 ± 0.04	0.46 ± 0.19	0.44 ± 0.12	0.35 ± 0.14	0.28 ± 0.04	0.22 ± 0.03	0.20

Bold value indicates variables which used to construct CDA functions.

Key environmental variables were underlined.

## Discussion

Pearson correlation showed a negative correlation between crab density and chlorophyll content and CCA results indicated that none of the fiddler crab species were correlated with chlorophyll content. However, CDA analysis (Fig. 4, Table 4) showed that chlorophyll content contributed in determining the habitat of *U*. *triangularis* (with the favorable range of 2.72 ± 0.67 μmol/g), *U*. *rosea* (2.07 ± 0.54 μmol/g), *U*. *forcipata* (4.42 ± 1.25 μmol/g) and *U*. *perplexa* (2.63 ± 0.55 μmol/g). This might be due to the negative correlation of chlorophyll content with carbon, nitrogen and total organic content, which were key factors in determining habitats of *U*. *triangularis*, *U*. *rosea*, *U*. *forcipata* and *U*. *perplexa*. Pearson correlation showed a positive relationship between chlorophyll content and pH and very fine sand. It seems that benthic microalgae tend to live in fine grained sediments with high amount of very fine sand (63μm) rather than sandy sediments. Besides, photosynthesis of benthic microalgae reduces carbon dioxide and consecutively increases pH in interstitial waters [23].

Heavy metals such as Cu, Zn, Ni, and Cr showed negative correlation with chlorophyll content. Photosynthetic activity of benthic microalgae created different layers of sediment based on redox potentials in mangrove muds [24]. In reduced sediments of mangrove forests, redox potential and pH are the main parameters controlling the accumulation of heavy metals [25–27]. The majority of heavy metals in sediment were found as metal sulfides [28,29]. Under reduced conditions, these metal compounds are stable. Once the oxidized zone develops due to the activity of benthic microalgae, metal sulfides will degenerate and metal ions will be released into the overlaying tidal water [28,29]. As the pH and redox potential were not significantly correlated with heavy metals in this study, it is unlikely to conclude that microalgae are responsible for low metal concentration. Indeed, the observed negative relationship between metals and chlorophyll content is simply the result of negative correlation between organic content and chlorophyll.

Salinity showed a positive correlation with sand particles (1mm, 250 μm, 125 μm). This could be related to lower water content, higher temperatures and consequently higher evaporation rates in sandy sediments. In sediments with high amount of sand particles (500, 250, 125, 63 μm), the porosity was lower than fine grained sediment. This feature makes these sandy substrates more compacted and denser with lower capacity to maintain interstitial water thus it is very likely to record higher temperature in these substrates during low tide. Fine grained sediment with high amount of silt-clay content retains more carbon, nitrogen and total organic contents. These sediments also contain more heavy metals than sandy sediment. Fine grained sediments have higher surface area, organic content and humic substances; thus have higher capacity to maintain heavy metals [30,31]. In this study, heavy metals showed positive relationship with organic content (Table 3). Ray et al., [32] found higher concentration of heavy metals in organic rich mangrove sediment in comparison with adjacent intertidal areas. Moreover, Marchand et al., [33] found positive correlation between Cr, Cu and Ni with total organic carbon. Positive correlation between organic matters and heavy metals is due to the presence of humic substances in organic matters which comprise 40 percent of sediment organic matters [34]. Humic substances are complex macromolecules created by humification process of biomolecules generated during decomposition of living organic matters [35]. Humic substances contain functional groups such as carboxyl and amino hydroxyl group which can bind with metal ions to form humic-metal complexes [36]. However, marine humates contain sulfur and nitrogen that also could bind with metal ions [34]. Nissenbaum and Swaine [37] suggested that humic substances can hold a large proportion of Cu, Mo and Zn, while sulfides are much important in combining with Ni and Co. Previous studies indicated a positive relationship between sulfur and organic content in mangrove sediments [33,38]. Sulfur minerals such as iron sulfide (FeS) and pyrite (FeS_2_) precipitate heavy metals in anoxic conditions [39]. Trace metals like Cd, Cu, Ni, Pb and Zn can replace Fe in iron sulfide and produce more stable compounds as trace metal mono sulfide [40].

**Fig 5:**
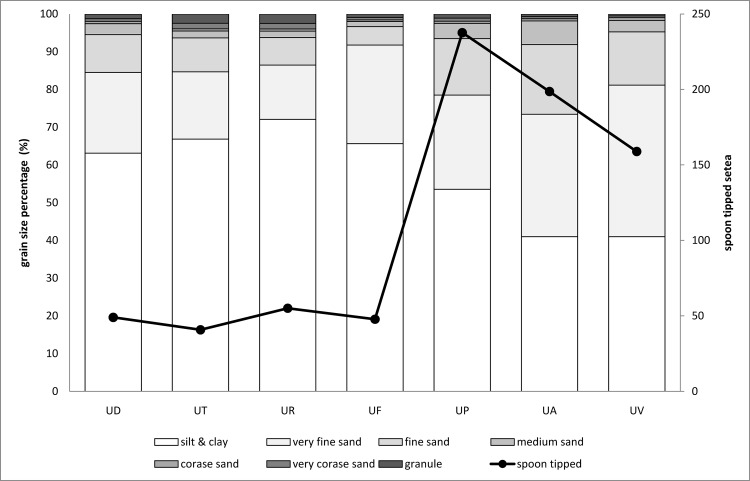
The percentage of grain size in sediment texture and mean number of spoon tipped setae in seven species of fiddler crabs with the average carapace width of 15mm. UD = *U*. *paradussumieri*, UT = *U*. *triangularis*, UR = *U*. *rosea*, UF = *U*. *forcipata*, UP = *U*. *perplexa*, UA = *U*. *annulipes*, UV = *U*. *vocans*.

CCA analysis showed that salinity and redox potential were negatively correlated with *U*. *paradussumieri*. Macintosh [41] stated that although *U*. *paradussumieri* have a similar range of salinity tolerance with other Malaysian fiddler crabs, they experience more metabolic cost when exposed to freshwater. Thus, *U*. *paradussumieri* normally avoid freshwater environment to reduce metabolic cost. However, they can commonly be found in low shore mudflats because they avoid high temperatures as well [42]. In lowest parts of the shore, sediment is too soft that only large individuals of *U*. *paradussumieri* can build and maintain burrows. In this part of the shore, porewater salinity varies greatly and reaches its minimum during the low tide due to higher freshwater discharge from the river. Negative correlation of *U*. *paradussumieri* with redox potential is due to the fact that low shore mudflats have less exposure time during the low tide. Therefore, low shore mudflats are less oxidized in comparison with high and mid shore mudflats probably because of lower concentration of oxygen in seawater comparing to air. On the low shore part of mud banks and in *U*. *paradussumieri* habitats, soluble nutrients like nitrate and ammonia were frequently washed away by tidal currents; therefore, lower nitrogen was observed in these sediments. This could justify the positive relationship between *U*. *Paradussumieri* and C:N ratio in Fig. 4. C:N ratio was a discriminant factor in determining the *U*. *paradussumieri*, *U*. *triangularis*, *U*. *rosea*, *U*. *forcipata* and *U*. *annulipes* habitats. *U*. *perplexa* and *U*. *annulipes* live in sandy sediments particularly with high amount of medium and fine sand (250 & 125 μm). The high number of spoon tipped setae in second maxiliped of *U*. *annulipes* and *U*. *perplexa* (Fig. 5) suggested that these species are specialized to feed on sandy sediments [43,44]. Edney [45] found that *U*. *annulipes* had a lower evaporative water loss and accordingly lower water requirement than other species. Table 4 indicated that fiddler crabs belong to *Austuca* subgenus (*U*. *annulipes*, *U*. *perplexa*, *U*. *triangularis*) usually live in mudflats with lower water content, which imply adaptation to live in semi-arid environments. *U*. *vocans* with a mean of 159 spoon tipped setae in their second maxiliped occupies habitat between muddy and sandy sediments with high amounts of very fine sand (grain size 63μm). In CCA plot (Fig. 3), *U*. *forcipata* showed positive correlation with mangrove trees, organic content, water content, porosity, silt-clay and negative correlation with temperature. It is common to find fine grained sediment with high amount of organic and carbon content near the mangrove trees where the *U*. *forcipata* lives. Edney [46] proposed that *U*. *forcipata* have lower tolerance to high temperature; therefore, they tend to live under the shadow of mangrove trees. Macintosh [41] stated that *U*. *rosea*, *U*. *triangularis*, *U*. *forcipata* and *U*. *paradussumier*i have a similar range of temperature tolerance but species which occupy low shore habitats such as *U*. *paradussumieri* and *U*. *forcipata* consume more energy for metabolic activity when they are exposed to high temperatures. *U*. *paradussumieri* avoids body heating by keeping their body moist with water reservoir in their burrows, while *U*. *forcipata* uses mangrove trees’ shade as a heat shelter. The low number of spoon tipped setae indicates that *U*. *forcipata* is specialized to feed on muddy sediments. The positive correlation between *U*. *forcipata* and Ni, Cr and Cu is because of the positive correlation between these elements and organic contents and fine grained sediment.

CCA results indicated that the *U*. *rosea* and *U*. *triangularis* habitats are very similar to each other. Such similarity could partly be explained by the sediment texture especially the percentage of very fine sand (63 μm). Both species live on the substrates with high sediment density and low amount of very fine sand (14–17%), while *U*. *forcipata* habitat contains 26% of very fine sand. Positive correlation of *U*. *rosea* and *U*. *triangularis* with nitrogen content might be related to the position of their habitat on high shore where the sediments are less exposed to tidal current and therefore they contain higher nitrogen. In the biplot of species and environmental parameters (Fig. 3B), the location of both *U*. *rosea* and *U*. *triangularis* points were in the opposite side of the very fine sand arrow. CDA results revealed that *U*. *rosea* and *U*. *triangularis* occupy mudflats with high sediment density. They were frequently seen on mudflats with sediment density above 8 g/ml (Table 4). It seems that these species unlike *U*. *paradussumieri* lack the ability to construct and maintain burrow in soft sediment. Macintosh [42] claimed that *U*. *rosea* and *U*. *triangularis* as high shore species possess a metabolic adaptation to resist high temperatures. Such adaptation gives them the ability to occupy high shore habitats where other species are scarcely found.

## Conclusion

The results of this study indicated that all environmental parameters except pH and redox potential were significantly correlated with sediment grain size. Sediment texture is the main factor which directly or indirectly influences sediment characteristics. Sediment texture is the major reason for sediment heterogeneity which creates different habitats for different species to occupy. Heavy metal concentration was low in all sampling stations and was not important in habitat determination of fiddler crabs. *U*. *paradussumieri* commonly live in low shore habitats where the amount of silt-clay and very fine sand comprised 85% of sediment texture. The number of spoon tipped setae confirmed that *U*. *paradussumieri* live on muddy substrate as feeding ground. Their habitat in low shore mud flats is too soft (with sediment density of 1. 69 g/ml) that only the large crabs could excavate and maintain burrows. The low number of spoon tipped setae in *U*. *forcipata* indicated that they adapted to feed on muddy shore. They were frequently found near the mangrove trees, where water, organic and carbon content is higher than other habitats. *U*. *rosea* and *U*. *triangularis* with the low number of spoon tipped setae (mean 55) occupy muddy sediments. These species construct their burrows in substrate with high sediment density. Because of their metabolic adaptation, they could resist high temperatures with no need of water chamber or mangrove tees as shelter. Consequently, they can be found in higher shore levels where discriminant factors like organic, carbon and nitrogen content are higher than these factors in low shore. *U*. *annulipes* and *U*. *perplexa* possess high numbers of spoon tipped setae in their second maxiliped. They live in sandy sediments with high sediment density and lower porosity, organic, carbon and nitrogen content in comparison with adjacent muddy substrates. *U*. *vocans* have moderate number of spoon tipped setae (159) and seems to live on sediments with higher amount of very fine sand (>40%). Environmental parameters controlling *U*. *vocans* distribution were however not determined in this study as CDA analysis cannot be applied to this species. This species was only found in one sampling station and more information is required from their distributed range in mangrove floor to define their habitat.
